# Development of a Molecular Serotyping Scheme for *Morganella morganii*

**DOI:** 10.3389/fmicb.2021.791165

**Published:** 2021-11-23

**Authors:** Bin Liu, Xi Guo, Jing Wang, Pan Wu, Shujie Li, Lu Feng, Bin Liu, Lei Wang

**Affiliations:** ^1^Tianjin Union Medical Center, TEDA Institute of Biological Sciences and Biotechnology, The Institute of Translational Medicine Research, Nankai University, Tianjin, China; ^2^Key Laboratory of Molecular Microbiology and Technology, Nankai University, Ministry of Education, Tianjin, China

**Keywords:** *Morganella morganii*, surface polysaccharide, O-antigen gene cluster, serotyping, microsphere-based suspension array

## Abstract

*Morganella morganii*, which is often regarded as a human commensal organism, can be an opportunistic pathogen, causing a variety of clinical infections with serious morbidity and mortality. An efficient and convenient method for subtyping and identifying *M. morganii* strains in epidemiological surveillance and control is urgently needed. Serotyping based on bacterial surface polysaccharide antigens (O-antigen or K-antigens) is a standard subtyping method for many gram-negative bacteria. Here, through whole genome sequencing and comparative genomics analysis of 27 strains, we developed a molecular serotyping scheme based on the genetic variation of O-antigen gene clusters (O-AGC) in *M. morganii*, and 11 distinct O-AGC types were identified. A conventional serotyping scheme was also developed by the production of antisera and agglutination experiments, which was shown to be perfectly consistent with the molecular serotyping scheme, confirming that the variation in *M. morganii* O-AGC correlated with phenotypic O-antigen diversification. Furthermore, a microsphere-based suspension array (MSA) with high specificity was developed based on the specific genes within each O-AGC type. The sensitivity of MSA was determined to be 0.1 ng of genomic DNA and 10^3^ CFU of pure culture. We further analyzed 104 *M. morganii* genomes available in GenBank, and an additional six novel O-AGC types were identified, indicating that the extension of this molecular serotyping scheme is convenient. Our work provides an important tool for the detection and epidemiological surveillance of *M. morganii*, and this method has the potential to be widely utilized, especially for bacterial genera/species without an efficient typing approach.

## Introduction

*Morganella morganii* is a Gram-negative bacillus belonging to the Enterobacteriaceae family, and it is a common inhabitant of the environment and intestinal tracts of humans, mammals, and reptiles (Erlanger et al., 2019). Conventionally, *M. morganii* is recognized as an unusual opportunistic pathogen that is isolated specifically in the urinary tract or wound infections. However, *M. morganii* has recently been regarded as an increasingly important pathogenic bacterium due to its virulence and increased drug resistance, which causes a variety of clinical infections, such as urinary tract infections, bacteremia and sepsis, and results in a high mortality rate in some infections (Liu et al., 2016; Minnullina et al., 2021).

Gram-negative bacteria produce lipopolysaccharide (LPS), which belongs to a family of structurally related glycolipids located in the outer membranes of bacteria (Megrian et al., 2020). A typical LPS molecule consists of a mix of well-conserved and highly variable structural elements (Supplementary Figure 1), including (i) lipid A, a component in the outer leaflet of the outer membranes that contributes to the barrier properties of bacteria and provides a pathogen-associated molecular pattern (Simpson and Trent, 2019); (ii) the core oligosaccharide (core OS), which is relatively conserved within a species, attaches to lipid A and is involved in the stability of the outer membrane (Whitfield and Trent, 2014); and (iii) the O-antigen or O-polysaccharide (O-PS), which is composed of hypervariable oligosaccharide repeating units (O-units) composed of two to eight different monosaccharide residues (heteroglycans) or identical sugars (homoglycans) in some bacteria (Valvano, 2003). The O-antigen is the most variable portion of LPS and provides the basis for serological specificity.

Serotyping based on the structural diversity of surface polysaccharide antigens, mainly O-antigen and K-antigen, remains the ‘gold standard’ for the detection and identification of strains, including pathogenic strains, in clinical specimens and environmental samples (Jacob et al., 2020; Elder et al., 2021). An O-antigenic scheme of *M. morganii* was established in the 1970s to 1990s (Vörös and Senior, 1990); however, the relevant antisera and the strains used for those studies are not currently available. The approach of traditional serotyping is limited by its high cost, high time and labor requirements, complicated procedures, cross-reactivity and subjective interpretation (Sumrall et al., 2020; Bian et al., 2021; Chaiden et al., 2021). To address these issues, many DNA-based serotyping methods on variant platforms targeting specific genes involved in the synthesis of surface polysaccharide antigens have been developed (Li et al., 2018, 2020; Zürn et al., 2020; Ouattara et al., 2021). In addition, a molecular serotyping scheme based on sequence variations of surface polysaccharide antigen synthesis genes has been rapidly established for several bacterial species. Moreover, the molecular serotyping scheme is theoretically consistent with the traditional serotyping system, and advances in next-generation sequencing have facilitated the establishment of these schemes (Sun et al., 2011; Guo et al., 2018).

Genes for O-antigen synthesis are normally clustered at the chromosome and named the O-AGC. Genetic variations in O-AGC are the major determinants of differences among the various O-antigens. The O-antigen synthesis genes fall into three major groups. The first group of genes is involved in the synthesis of nucleotide sugar precursors of the O-antigen. The second group, glycosyltransferase genes, is responsible for the sequential transfer of precursor sugars to undecaprenyl phosphate (UndP), thus forming the UndPP-O-unit. The O-antigen processing proteins, which are encoded by genes of the third group (*wzx*/*wzy* or *wzm*/*wzt*), are involved in translocation across the membrane and polymerization of the O-unit (Samuel and Reeves, 2003). According to the assembly mechanism, the process of O-antigen synthesis can be divided into three major pathways: the Wzx/Wzy-dependent pathway, which is usually used for heteroglycan synthesis (Islam and Lam, 2014), the ABC transporter (encoded by *wzm* and *wzt*) pathway, which is common during homoglycan synthesis (Greenfield and Whitfield, 2012), and the synthase pathway that has only been reported in *Salmonella enterica* O54 (Keenleyside et al., 1994). O-antigen processing genes and glycosyltransferase genes are always specific to individual O-antigens, and they are used in molecular assays to detect strains belonging to different serogroups (Ballmer et al., 2007).

*Morganella morganii* belongs to the tribe Proteeae along with another two members, *Proteus* and *Providencia*. Currently, no DNA-based typing assay, such as multilocus sequence typing (MLST), has been developed in *M. morganii*. Our group has investigated the O-AGCs of *Proteus* and *Providencia* in depth and developed molecular serotyping systems for each (Yu et al., 2017; Du et al., 2018). To our knowledge, the location of O-AGC in the genome of *M. morganii* remains unclear. Here, by sequencing and comparatively analyzing the genomes of 27 *M. morganii* strains from Shanghai Disease Control and Prevention, China, we established a molecular serotyping scheme for *M. morganii* with 11 different putative O-AGC types. The production of antisera against each O-AGC type and agglutination experiments confirmed the accuracy of our molecular serotyping scheme. A MSA based on O-AGC-specific genes was also developed. In summary, our work provides a promising and efficient tool for molecular diagnostics and epidemiological surveillance of *M. morganii*.

## Materials and Methods

### Genomic Sequencing and Bioinformatic Analysis

Twenty-seven *M. morganii* strains (Table 1) were cultured overnight in Luria-Bertani broth at 37°C with shaking. Subsequently, genomic DNA was extracted from 1.5 mL of each of the overnight bacterial cultures using a DNA extraction kit according to the manufacturer’s instructions (Tiangen, Beijing, China). The genomic DNA was sheared, polished, and prepared using the Illumina Sample Preparation Kit. Genomic libraries containing 500-bp paired-end inserts were constructed, and sequencing was then performed with Solexa sequencing technologies (Illumina Inc., San Diego California, United States) to produce approximately 100-fold coverage. The obtained reads were assembled using the *de novo* genome assembly program Velvet to generate a multicontig draft genome. Next, Artemis (Rutherford et al., 2000) was subjected to annotate genes. BLAST and PSI-BLAST were used to search available databases, including the GenBank^1^ and Pfam protein motif databases (pfam.sanger.ac.uk), for gene and protein annotation (Mistry et al., 2021), respectively. The TMHMM v2.0 analysis program^2^ was used to identify potential transmembrane domains within the protein sequences.

**TABLE 1:** Strains used in this study and their allocation based on their O-AGC type.

**O-AGC**	**Strains**
Type 1	**G6338**, G6355, G6369
Type 2	**G6341**, G6339
Type 3	**G6342**, G6346
Type 4	**G6345**, G6362, G6350, G6356
Type 5	**G6352**, G6340, G6348, G6363, G6344, G6343
Type 6	**G6354**
Type 7	**G6359**
Type 8	**G6360**, G6349, G6361
Type 9	**G6364**
Type 10	**G6367**
Type 11	**G6368**, G6365, G6366

*Type strains of each O-AGC are indicated in bold.*

### Preparation of Antigens and Antisera

All strains used for immunization were cultured in 10 mL of Luria-Bertani agar overnight. The cultures were harvested by centrifugation at 5,000 × g for 20 min, washed in 20 mL of 0.85% NaCl, and suspended in 20 ml of 0.85% NaCl (approximately 10^9^ cells/ml). The cell suspensions were subsequently heated at 121°C for 30 min and then cooled. Only strains that did not exhibit autoagglutination were used as the resultant antigens for immunization. Subsequently, adult New Zealand White rabbits (12 weeks of age) were injected intravenously with the prepared antigens. At 3-day intervals, injections were administered at doses of 0.5 ml, 1 ml, 2 ml, and 4 ml. One week after the final injection, the rabbit was exsanguinated, and the separated antisera were stored at 4°C.

### Agglutination Test and Antisera Absorption

Antigens were prepared from the cells cultured overnight in 10 mL of Luria-Bertani agar at 37°C and then mixed with 25 μL of 2-fold diluted antiserum in 0.85% NaCl. The selected titer was the most diluted concentration of antiserum that gave a positive reaction. The cell suspensions used for absorption were washed three times with 0.85% NaCl and resuspended in 3 mL of antiserum. The mixture was incubated at room temperature for 2 h and then centrifuged at 10,000 × g for 15 min, after which the supernatant was collected. The absorbed antiserum was tested against all antigens that reacted with the unabsorbed antiserum. This process was repeated until cross-reactions no longer occurred.

### Development of a Microsphere-Based Suspension Array for Molecular Serotyping

Almost all primers and probes (Table 2) for MSA were designed based on the *wzx* or *wzy* gene using Primer Premier v5.0 software (Premier Biosoft International, Palo Alto, CA, United States), except those targeting *orf10*_type__8_, which is utilized to differentiate type 8 from type 5. Multiplex PCR amplification was performed in a 50 μL reaction mixture composed of 100 ng genomic DNA, 1 × Goldstar PCR buffer, 0.04 mM deoxynucleoside triphosphates, 0.1 mM each primer, and 1 unit Goldstar DNA polymerase. The PCR parameters were as follows: 95°C for 10 min; 30 cycles at 98°C for 10 s, 55°C for 30 s, and 72°C for 30 s; and a final extension at 72°C for 5 min. Probe-microsphere coupling, hybridization and MSA analysis were performed as described previously (Guo et al., 2018). A positive signal was defined as a median fluorescence intensity (MFI) ≥ 200 and a signal/background ratio (S/B ratio = MFI/Blank) > 5.

**TABLE 2:** Primers and probes used for the multiplex Luminex-based assay.

**O-AGC**	**Target gene**	**Primer sequence (5′-3′)**		**Probe sequence^b^**
		**Forward**	**Reverse^a^**	
Type 1	*wzx*	wln-29839: TTCATTGTCGGCACATTCAGT	wln-29840: AAAGCGGGATACCCGAAGT	OAn-983: AATCTATGGCTCTCAATATCATG
Type 2	*wzy*	wln-29841: ACCTCATTTGACACCTCGTTTG	wln-29842: GAAGTACACCAATTAAACCCAGTTC	OAn-965: TTTGGCGCAGGCCCTGGTG
Type 3	*wzx*	wln-29843: TGCTCGTGCCAGCGTATC	wln-29844: AAATAAAGCCGACTACAGCTCCTA	OAn-976: CCTAGGTATCTTCTCTGGTTC
Type 4	*wzy*	wln-29845: TTCTCCAGCAGTTAGCCT	wln-29846: CAAAGCGTAGTCCTGATG	OAn-967: TTCCTCAAGCTAGATAATGATA
Type 5/8	*wzx*	wln-29847: ACTCGCCGTTCTCGGATTAT	wln-29848: TCATGTGACTCATTGACCCACTC	OAn-977: GGTAAAGTAAGAAGCAACAGATT
Type 8	*orf10*	TGAAAATAAATGATGAGC	TCACCAAATGATACACCC	OAn-982: AAATGGGAAAAAATAAGA
Type 6	*wzx*	wln-29849: ATATTGGTGGCTTGGTTCTGTT	wln-29850: AATACATAGTCAGGATGTGCTCCAT	OAn-978: CCTTTCAGGCCACAGGA
Type 7	*wzx*	wln-29853: ACCTTATTACGCTTATGCTGTGG	wln-29854: GAGGATGCAAATCCATTTACGA	OAn-980: CACTTGGTCAATTACAAAGTT
Type 9	*wzx*	wln-29855: TTTAGCAAAACTACTTACCCTCCC	wln-29856: GCTTGGGCAATAGGGTTCA	OAn-981: TAAGCACTCCGAATGTTTG
Type 10	*wzx*	wln-29857: TACGGCTTATCGGGTGCTC	wln-29858: CCATAATCCTTGCCAATACCC	OAn-973: TCTCATTGCACTAGCAACTAACCAATCGATT
Type 11	*wzx*	wln-29859: GAAAATACTGGCAACTCAAGCTC	wln-29860: TGGTGCCGAAATAGTGGAATA	OAn-990: GATTGGTTGTTTACCTATGC

*^a^Each reverse primer was labeled with biotin at the 5**′** end.*

*^b^Each probe was synthesized with an amino C-12 module at the 5**′** end.*

## Results

### Location and Genetic Features of O-Antigen Gene Clusters in *Morganella morganii*

By sequencing and analyzing the genomes of 27 *M. morganii* isolates, several putative gene clusters associated with polysaccharide synthesis were annotated (data not shown). Among them, a genetic region between two housekeeping genes was analyzed, and the locus of each isolate shares some common features. First, all of the loci are mapped between two flanked genes, *cpxA* and *secB*, which encode a two component system sensor kinase and protein-export chaperone, respectively, as in the case of *Proteus* O-AGC (Yu et al., 2017; Du et al., 2018). Second, each locus contains three classes of genes associated with O-antigen synthesis. Third, each locus shows a lower % GC content (∼35%) than that of the whole genome (∼51%) (Chen et al., 2012). Therefore, we propose that the genetic region between *cpxA* and *secB* is a candidate O-AGC for *M. morganii*, and it may also be recently obtained by lateral transfer from a different species, as in many other bacteria.

**FIGURE 1:**
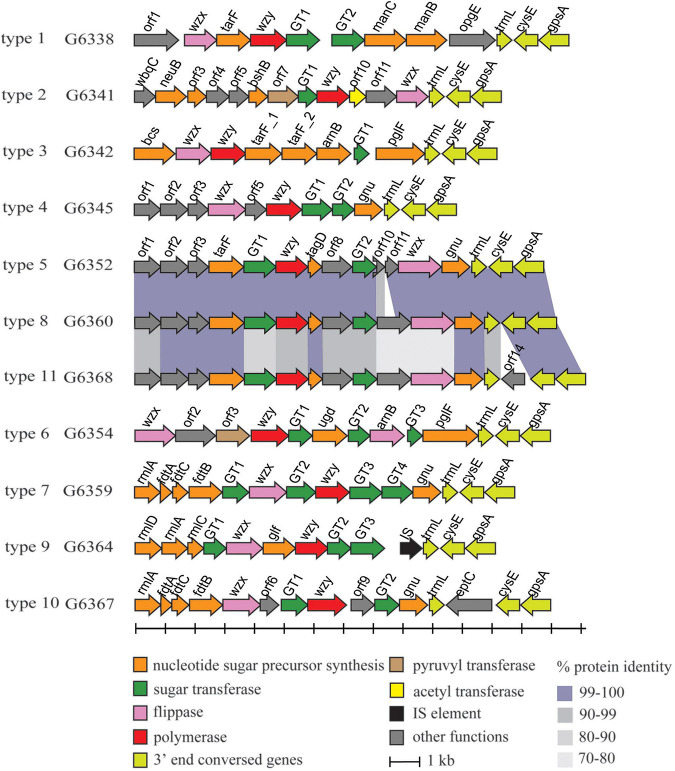
Schematic diagram of the 11 putative O-AGCs characterized from the 27 *M. morganii* strains. Genes are represented by arrows and colored according to the gene key at the bottom.

In total, 11 types of putative O-AGCs were identified, with their nucleotide sequences ranging in size from 10,850 to 15,182 bp. A conserved gene set *trmL-cysE-gpsA* is located at the 3′ end of each O-AGC, with the minor exceptions of one gene being inserted between *trmL* and *cysE* in type 10 and type 11. Moreover, the *wzx* gene encoding O-unit flippase and the *wzy* gene encoding O-antigen polymerase were both found within each O-AGC, indicating that *M. morganii* probably synthesizes the O-antigen via the Wzx/Wzy-dependent pathway (Figure 1). The characteristics of open reading frames (ORFs) for all putative O-AGCs are summarized in Supplementary Table 1.

Several sugar residues within the O-unit of *M. morganii* can be predicted based on the presence of corresponding nucleotide sugar precursor synthesis genes in O-AGC. For instance, the *manC* gene encoding mannose-1-phosphate guanylyltransferase and the *manB* gene encoding phosphomannomutase were assigned to O-AGC type 1, and their products are involved in the synthesis of GDP-D-mannose along with phosphomannose isomerase ManA (*manA* gene is always outside the O-AGC) (Samuel and Reeves, 2003). In O-AGC type 9, *rmlD/A/C* and *glf* were annotated. RmlA/B/C/D have been identified as responsible for the synthesis of dTDP-L-rhamnose, the nucleotide sugar precursor of L-rhamnose (Allard et al., 2001), and Glf (UDP-galactopyranose mutase) has been identified to catalyze the conversion from UDP-galactose pyranose to UDP-galactose furanose (Nassau et al., 1996). In O-AGC types 7 and 10, a *rmlA/ftdA/C/B* set is located at the 5’ region. The enzymes encoded by these four genes (glucose-1-phosphate thymidylyltransferase, dTDP-6-deoxy-hex-4-ulose isomerase, dTDP-6-deoxy-D-xylo-hex-3-ulose aminase and dTDP-D-Fuc3N acetylase), together with dTDP-D-glucose 4,6-dehydratase (encoded by *rmlB* gene), have been identified to be involved in the synthesis of dTDP-3-acetamido-3-deoxy-D-fucose (dTDP-Fuc3NAc) (Pfoestl et al., 2003). It should be noted that *rmlB*, which is always present along with *rmlACD* genes, is not found within O-AGC 9, 7, and 10, thus indicating that *rmlB* may be located elsewhere at the chromosome, and we successively assigned the homolog of *rmlB* in the genome of strains belonging to O-AGC types 9, 7, and 10.

In addition, several O-AGCs of *M. morganii* are genetically related. For instance, the sequences of type 5 and type 8 O-AGCs share high-level identity (96 to 100%) except for the genes between GT2 and *wzx*, respectively. In addition, the O-AGCs of type 8 and type 11 also share high level identity (72 to 100%), with the exception of *orf14* inserted between *trmL* and *cysE* of type 11 O-AGC. Compared with the nucleotide sugar precursor synthesis genes, the identity of glycosyltransferase genes and processing genes (*wzx/wzy*) between these two O-AGCs was relatively low (Figure 1), indicating that the products encoded by these genes may determine the specificity of the O-antigen structure of these two types.

Collectively, these data suggest that the region between *cpxA* and *secB* in the *M. morganii* genome exhibits the genetic features of O-AGC and a potential molecular serotyping scheme could be established in *M. morganii* based on the genetic diversity of O-AGC types.

### Conventional Serotyping Scheme Is Correlated Well With the Molecular Serotyping Scheme

Antiserum against each type strain representing unique O-AGC was prepared, and an agglutination test was performed. The homologous and heterologous titers are summarized in Table 3. Generally, each antiserum reacted to its homologous strain with high titers (160 to 320). Simultaneously, except for the antiserum against G6352, G6354, G6359, and G6364, the remainders agglutinated with heterologous strains and thus needed to be absorbed. After absorption, all the cross reactions disappeared and each absorbed antiserum only generated a positive result with its corresponding homologous strain. Next, we tested 16 other strains with the absorbed antisera. As expected, each of them reacted with only one of the 11 antisera, and the grouping is consistent with the case in the O-AGC allocation. These data suggest that the conventional serotyping scheme is perfectly consistent with the molecular serotyping scheme, thus confirming that the variation in *M. morganii* O-AGC is correlated with phenotypic O-antigen diversification. We designated G6338, G6341, G6342, G6345, G6352, G6354, G6359, G6360, G6364, G6367, and G6368 as type strains (types 1 to 11) for each O-AGC type.

**TABLE 3:** Homologous and heterologous agglutinin titers of antisera before absorption.

**Antiserum to strain**	**Agglutinin titers to strain**										
	**G3668**	**G6341**	**G6342**	**G6345**	**G6352**	**G6354**	**G6359**	**G6360**	**G6364**	**G6367**	**G6368**
G3668	320					80					
G6341		160				20			40		
G6342			160			80			40		
G6345				160		40			80		
G6352					320						
G6354						320					
G6359							320				
G6360						80		320			
G6364									320		
G6367						160			160	320	
G6368					40	40					320

### Development of a Microsphere-Based Suspension Assay

**FIGURE 2:**
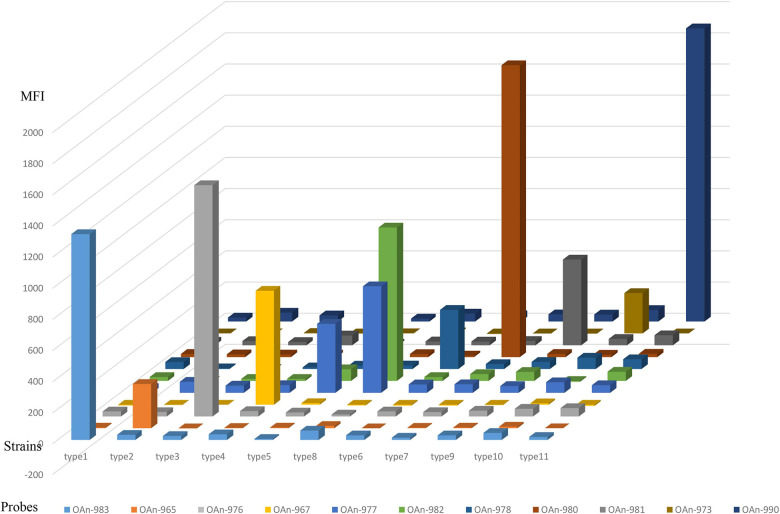
Specific test of the MSA assay. The hybridization signals are presented in terms of median fluorescence intensity (MFI) on the *y*-axis, and each sample representing the corresponding type strain is indicated on the *x*-axis. Probe OAn-982 were designed targeting *orf10*_type__8_ to differentiate type 8 from type 5.

Compared to the nucleotide sugar precursor synthesis genes and glycosyltransferase genes, O-antigen processing genes are always more heterogeneous (Ballmer et al., 2007). Thus, the *wzx* or *wzy* gene was used for primer and probe design in our study, and *orf10*_type__8_ was selected to differentiate type 8 from type 5. The 27 *M. morganii* strains carrying O-AGC 1 to 11 and other bacteria that are genetically close to *M. morganii* or frequently isolated from the urinary tract, including *Proteus spp*. (*n* = 5), *Providencia spp*. (*n* = 4), *Escherichia coli* (*n* = 2), *Klebsiella pneumoniae* (*n* = 2), *Enterococcus faecalis* (*n* = 2), Group B *Streptococcus* (*n* = 1), and *Staphylococcus aureus* (*n* = 1), were used to test the specificity of the microsphere-based suspension (MAS) assay. Our test showed that each O-AGC-specific probe detected the homologous strains correctly and no hybridization signals were generated against the heterologous strains, indicating the accurate specificity of our assay (Figure 2). Moreover, a sensitivity test was performed using genomic DNA or pure cultures from the type strains containing each O-AGC type. Generally, the genomic DNA of each type strain was serially diluted by 10-fold (10 ng to 0.1 pg) as the template, and the target could be determined using at least 0.1 ng DNA. Our results also showed that the MAS array could generate a positive signal for boiled supernatants from at least 10^3^ CFU of pure bacterial cultures.

### Serotype Allocation Using Genome Data

A program was designed by us to perform the *in silico* serotyping of *M. morganii* using the genome data. In brief, a database was first generated based on the *wzx* or *wzy* genes and *orf10*_type__8_, which were all verified by our MSA assay. Next, the 104 *M. morganii* genome assemblies from GenBank were subjected to a BLASTn search against the database with a coverage cutoff of >90% and an identity cutoff of >99%. Among them, 42 strains could be assigned to one of the eight O-AGC types characterized here, with type 8 (43%) and type 11 (26%) representing the predominant groups, and none of the strains matched to types 2, 4 and 10 (Supplementary Table 2).

**FIGURE 3:**
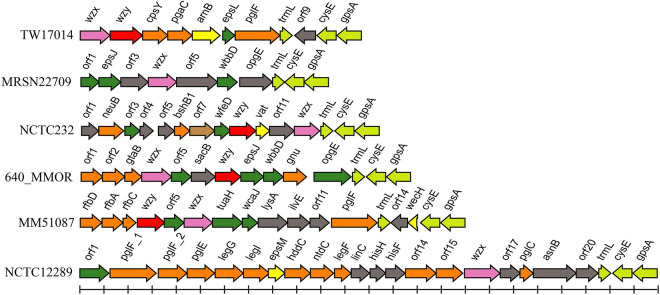
Schematic diagram of the six temporary O-AGCs extracted from *M. morganii* genomes from GenBank. For the gene key, see Figure 1.

Among the remaining 62 genome assemblies, 16 were too fragmented and were thus excluded from the next analysis. The genetic regions between *cpxA* and *secB* in 46 strains were extracted and analyzed, and six additional putative O-AGCs (temp1 to 6) were characterized (Figure 3 and Supplementary Table 3).

## Discussion

According to a recent classification based on genomic phylogeny, *Morganella* is a type genus of the novel Morganellaceae family, which also includes the genetically adjacent genera *Proteus* and *Providencia* (Adeolu et al., 2016). The sharing of the same O-AGC locus by *Morganella* and *Proteus* also indicates the close relationship between the two genera. Several methods have been developed for *M. morganii* subclassification, including biotyping (Rauss and Vörös, 1959), phage typing (Schmidt and Jeffries, 1974), bacteriocin typing (Senior, 1987), and protein profile typing (Senior and Vörös, 1990). Among these techniques, biotyping and phage typing have low distinguishing power. Although much more discriminating, the typing method by bacteriocin testing and protein profiling is time-consuming and laborious. Currently, whole-genome sequencing (WGS)-based approaches, mainly including core genome multilocus sequence typing (cg-MLST) and single-nucleotide polymorphism (SNP)-based approaches, have been presented and applied to pathogenic epidemiological surveillance, thus providing maximal discrimination compared to other methods (Hazen et al., 2016; Njamkepo et al., 2016; Beyrouthy et al., 2018). However, the WGS-based approach has not been applied widely to routine detection, especially in basic infection control agencies, due to its relatively high cost and lengthy turnaround time.

The variation in O-antigen structures provides the basis for serotyping many Gram-negative bacteria, and it has been widely used to classify strains for epidemiological investigation and surveillance and represents the ‘gold standard’ (Liu et al., 2020). Conventional serotyping based on agglutination reactions is also limited by the high prevalence of non-typeable isolates and cross reactions, which is common in clinical isolates. The molecular serotyping system developed by our group correlated well with the traditional antigenic scheme and can offer better resolution (Guo et al., 2018). Accordingly, we believe that fast and reliable PCR-based methodologies targeting serospecific genes, such as MSA technology, are ideal for pathogenic classification and epidemiological purposes.

Among six novel putative O-AGCs, no *wzy* gene was assigned in strains MRSN22709 and NCTC12289 (Figure 3). It is likely that the functional *wzy* gene is located outside the O-AGC in these two strains. This atypical feature has been reported in other species. For example, the *wzy* gene maps far from the O-AGCs in *Salmonella* serogroups A, B, and D1 (Wang et al., 2002). By screening the genome assemblies, we also observed that the *cpxA*-*secB* regions in five genomes possess neither glycosyltransferase genes nor possessing genes. It is likely that in these isolates, the functional O-AGCs reside in other sites of the chromosome, which will be the subject of future studies.

The chemical structure of *M. morganii* O-antigens should be elucidated in the future to further support our findings. In addition, as O-antigen is an important virulence factor associated with bacterial pathogenesis (March et al., 2013; Sarkar et al., 2014; Caboni et al., 2015), our work also provides the basis for future studies on the role of O-antigen in *M. morganii* pathogenesis and could lead to a deeper understanding of the virulence mechanisms of this bacterium.

## Data Availability Statement

The data presented in the study are deposited in the GenBank repository, accession numbers OK482582–OK482592.

## Ethics Statement

The animal study was reviewed and approved by Institutional Animal Care Committee at Nankai University (Tianjin, China; protocol code 20190325.02).

## Author Contributions

LW and BL (correspondence) designed the research. BL (first author), XG, JW, PW, and SL performed the research. LF provided technical support and insights. XG and JW analyzed the data. XG and BL (correspondence) wrote the manuscript. All authors contributed to the article and approved the submitted version.

## Conflict of Interest

The authors declare that the research was conducted in the absence of any commercial or financial relationships that could be construed as a potential conflict of interest.

## Publisher’s Note

All claims expressed in this article are solely those of the authors and do not necessarily represent those of their affiliated organizations, or those of the publisher, the editors and the reviewers. Any product that may be evaluated in this article, or claim that may be made by its manufacturer, is not guaranteed or endorsed by the publisher.

## Footnotes

1
www.ncbi.nlm.nih.gov/genbank

2
http://www.cbs.dtu.dk/services/TMHMM-2.0/
