# Supplementary material for: Development of a Molecular Serotyping Scheme for Morganella morganii
Source: Front Microbiol. 2021 Nov 23;12:791165. doi: 10.3389/fmicb.2021.791165 (PMC8649690; doi:10.3389/fmicb.2021.791165)
Supplement: Supplementary file 1 [file Table_1.DOC]

Supplementary Table 1. Characteristics of the ORFs in 11 *Morganella morganii* O-antigen gene clusters

G6338 (type 1)

| Orf no. | Gene name | Position of gene | G+C content(%) | Similar protein(s), strain(s) (Genbank accession No.) | %Identical/%Similar (total No. of aa) | Putative function of protein |
| --- | --- | --- | --- | --- | --- | --- |
| 1 | *orf1* | 1..1494 | 28.38 | HAD-IIA family hydrolase [Morganella morganii]([WP_163654150.1](https://www.ncbi.nlm.nih.gov/protein/WP_163654150.1?report=genbank&log$=protalign&blast_rank=1&RID=ZZPDG17S013)) | 99/99（497） | Acid sugar phosphatase |
| 2 | *wzx* | 1543..2781 | 25.64 | oligosaccharide flippase family protein [Morganella morganii]  ([WP_163654153.1](https://www.ncbi.nlm.nih.gov/protein/WP_163654153.1?report=genbank&log$=protalign&blast_rank=1&RID=ZZPHKZ7X013)) | 96/97（408） | Putative O-antigen transporter |
| 3 | *tarF* | 2774..3919 | 23.99 | CDP-glycerol glycerophosphotransferase family protein, partial [Morganella morganii]([WP_176211890.1](https://www.ncbi.nlm.nih.gov/protein/WP_176211890.1?report=genbank&log$=protalign&blast_rank=2&RID=ZZPN9NP6013)) | 90/97（156） | Teichoic acid poly(glycerol phosphate) polymerase |
| 4 | *wzy* | 3919..5133 | 23.62 | hypothetical protein [Morganella morganii]([MQC08413.1](https://www.ncbi.nlm.nih.gov/protein/MQC08413.1?report=genbank&log$=protalign&blast_rank=1&RID=ZZPSZHFD013)) | 46/63（404） | polymerase |
| 5 | *GT1* | 5123..6238 | 28.04 | glycosyltransferase [Morganella morganii] ([WP_163654158.1](https://www.ncbi.nlm.nih.gov/protein/WP_163654158.1?report=genbank&log$=protalign&blast_rank=1&RID=ZZPWNW8R016)) | 94/98（371） | glycosyltransferase |
| 6 | *GT2* | 6654..7760 | 29.08 | glycosyltransferase [Escherichia coli] ([WP_135405073.1](https://www.ncbi.nlm.nih.gov/protein/WP_135405073.1?report=genbank&log$=protalign&blast_rank=2&RID=ZZR091RT016) ) | 64/79（368） | GDP-mannose-dependent alpha-(1-6)-phosphatidylinositol monomannoside  mannosyltransferase |
| 7 | *manC* | 7761..9158 | 33.9 | mannose-1-phosphate guanylyltransferase/mannose-6-phosphate isomerase [Morganella morganii] ([WP_152696934.1](https://www.ncbi.nlm.nih.gov/protein/WP_152696934.1?report=genbank&log$=protalign&blast_rank=1&RID=ZZR44W8G016)) | 88/95（465） | Mannose-1-phosphate guanylyltransferase RfbM |
| 8 | *manB* | 9161..10525 | 36.77 | phosphomannomutase CpsG [Morganella morganii]  [MQC08417.1](https://www.ncbi.nlm.nih.gov/protein/MQC08417.1?report=genbank&log$=protalign&blast_rank=1&RID=ZZR82N99013) | 96/98（455） | Phosphomannomutase/phosphoglucomutase |
| 9 | *opgE* | 10620..12161 | 36.9 | phosphoethanolamine transferase [Morganella morganii]   ([WP_152696932.1](https://www.ncbi.nlm.nih.gov/protein/WP_152696932.1?report=genbank&log$=protalign&blast_rank=2&RID=ZZRBURE6013)) | 96/97（513） | Phosphoethanolamine transferase OpgE |
| 10 | *trmL* | 12215..12718 | 50.39 | tRNA(uridine(34)/cytosine(34)/5-carboxymethylaminomethyluridine(34)-2'-O)-methyltransferase TrmL [Morganella morganii]  ([WP_025152359.1](https://www.ncbi.nlm.nih.gov/protein/WP_025152359.1?report=genbank&log$=protalign&blast_rank=1&RID=ZZRGUCV2016)) | 100/100（167） | tRNA (cytidine(34)-2'-O)-methyltransferase |
| 11 | *cysE* | complement(12792..13592) | 55.43 | serine O-acetyltransferase [Morganella morganii]   ([WP_064483679.1](https://www.ncbi.nlm.nih.gov/protein/WP_064483679.1?report=genbank&log$=protalign&blast_rank=2&RID=ZZRM0TCV01R)) | 100/100（267） | Serine acetyltransferase |
| 12 | *gpsA* | complement(13626..14639) | 56.7 | NAD(P)H-dependent glycerol-3-phosphate dehydrogenase [Morganella morganii] ([WP_163654164.1](https://www.ncbi.nlm.nih.gov/protein/WP_163654164.1?report=genbank&log$=protalign&blast_rank=2&RID=ZZRR3G3001R)) | 99/100（337） | Glycerol-3-phosphate dehydrogenase [NAD(P)+] |

G6341 (type 2)

| Orf no. | Gene name | Position of gene | G+C content(%) | Similar protein(s), strain(s) (Genbank accession No.) | %Identical/%Similar (total No. of aa) | Putative function of protein |
| --- | --- | --- | --- | --- | --- | --- |
| 1 | *wbqC* | 1..711 | 25.31 | WbqC family protein [Morganella morganii] ([WP_015422389.1](https://www.ncbi.nlm.nih.gov/protein/WP_015422389.1?report=genbank&log$=protalign&blast_rank=1&RID=ZZRWS8B501R)) | 100/100（236） | hypothetical protein |
| 2 | *neuB* | 711..1751 | 34.29 | N-acetylneuraminate synthase family protein [Morganella morganii]([WP_004236628.1](https://www.ncbi.nlm.nih.gov/protein/WP_004236628.1?report=genbank&log$=protalign&blast_rank=1&RID=ZZS133KE013)) | 100/100（346） | N,N'-diacetyllegionaminic acid synthase" |
| 3 | *ofr3* | 1796..2428 | 35.07 | acylneuraminate cytidylyltransferase [Candidatus Altiarchaeales archaeon HGW-Altiarchaeales-2] (PKP60156.1) | 37/57（210） | acylneuraminate cytidylyltransferase |
| 4 | *orf4* | 2425..3195 | 31.25 | hypothetical protein [Morganella morganii]( WP_015422387.1) | 100/100（256） | hypothetical protein |
| 5 | *orf5* | 3188..3856 | 28.4 | hypothetical protein [Morganella morganii]([WP_015422386.1](https://www.ncbi.nlm.nih.gov/protein/WP_015422386.1?report=genbank&log$=protalign&blast_rank=1&RID=ZZSC79Z4016)) | 100/100（222） | hypothetical protein |
| 6 | *bshB* | 3853..4494 | 32.86 | PIG-L family deacetylase [Morganella morganii]   ([WP_004236630.1](https://www.ncbi.nlm.nih.gov/protein/WP_004236630.1?report=genbank&log$=protalign&blast_rank=1&RID=ZZSFN61Z013)) | 100/100（213） | N-acetyl-alpha-D-glucosaminyl L-malate deacetylase |
| 7 | *orf7* | 4491..5528 | 28.51 | polysaccharide pyruvyl transferase family protein [Morganella morganii] ([WP_015422385.1](https://www.ncbi.nlm.nih.gov/protein/WP_015422385.1?report=genbank&log$=protalign&blast_rank=2&RID=ZZSM16TW013)) | 99/100（343） | Polysaccharide pyruvyl transferase |
| 8 | *GT1* | 5518..6183 | 29.12 | WecB/TagA/CpsF family glycosyltransferase [Morganella morganii] ([WP_015422384.1](https://www.ncbi.nlm.nih.gov/protein/WP_015422384.1?report=genbank&log$=protalign&blast_rank=1&RID=ZZWAA2XJ016)) | 100/100（221） | UDP-Gal:alpha-D-GlcNAc-diphosphoundecaprenol beta-1,4-galactosyltransferase |
| 9 | *wzy* | 6146..7240 | 28.4 | hypothetical protein MU9_558 [Morganella morganii subsp. morganii KT]([AGG29604.1](https://www.ncbi.nlm.nih.gov/protein/AGG29604.1?report=genbank&log$=protalign&blast_rank=1&RID=ZZWGVJJN013)) | 100/100（364） | polymerase |
| 10 | *orf10* | 7244..7810 | 32.27 | CatB-related O-acetyltransferase [Acinetobacter baumannii] (WP_098714275.1 ) | 40/57（165） | O-acetyltransferase |
| 11 | *orf11* | 7800..8831 | 27.9 | hypothetical protein [Morganella morganii] ([WP_152902692.1](https://www.ncbi.nlm.nih.gov/protein/WP_152902692.1?report=genbank&log$=protalign&blast_rank=2&RID=ZZWU388C016)) | 99/99（343） | hypothetical protein |
| 12 | *wzx* | 8828..9889 | 22.88 | hypothetical protein [Morganella morganii] ([WP_015422381.1](https://www.ncbi.nlm.nih.gov/protein/WP_015422381.1?report=genbank&log$=protalign&blast_rank=1&RID=ZZWXDGVY016)) | 99/99（353） | flippase |
| 13 | *trmL* | 9929..10432 | 50.19 | tRNA(uridine(34)/cytosine(34)/5-carboxymethylaminomethyluridine(34)-2'-O)-methyltransferase TrmL [Morganella morganii]   ([WP_025152359.1](https://www.ncbi.nlm.nih.gov/protein/WP_025152359.1?report=genbank&log$=protalign&blast_rank=2&RID=ZZX0JZU101R)) | 99/99（167） | tRNA (cytidine(34)-2'-O)-methyltransferase |
| 14 | *cysE* | complement(10506..11306) | 55.43 | serine O-acetyltransferase [Morganella morganii]   ([WP_004236641.1](https://www.ncbi.nlm.nih.gov/protein/WP_004236641.1?report=genbank&log$=protalign&blast_rank=2&RID=ZZX3GN7P01R)) | 100/100（266） | Serine acetyltransferase |
| 15 | *gpsA* | complement(11341..12354) | 57.1 | NAD(P)H-dependent glycerol-3-phosphate dehydrogenase [Morganella morganii]([WP_163654164.1](https://www.ncbi.nlm.nih.gov/protein/WP_163654164.1?report=genbank&log$=protalign&blast_rank=2&RID=ZZX6BC4T01R)) | 99/100（337） | Glycerol-3-phosphate dehydrogenase [NAD(P)+] |

G6342 (type 3)

| Orf no. | Gene name | Position of gene | G+C content(%) | Similar protein(s), strain(s) (Genbank accession No.) | %Identical/%Similar (total No. of aa) | Putative function of protein |
| --- | --- | --- | --- | --- | --- | --- |
| 1 | *bcs* | 1..1377 | 32.67 | 2-C-methyl-D-erythritol 4-phosphate cytidylyltransferase [Morganella morganii] ([WP_126324183.1](https://www.ncbi.nlm.nih.gov/protein/WP_126324183.1?report=genbank&log$=protalign&blast_rank=1&RID=ZXPFFF5M016)) | 100/100(458) | Bifunctional ribulose 5-phosphate reductase |
| 2 | *wzx* | 1383..2597 | 30.11 | Wzx [Proteus vulgaris] (AXY99856.1) | 63/100(404) | flippase |
| 3 | *wzy* | 2594..3766 | 25.23 | Wzy [Proteus vulgaris] (AXY99857.1) | 58/72(390) | polymerase |
| 4 | *tarF_1* | 3741..4970 | 25.85 | CDP-glycerol glycerophosphotransferase family protein [Morganella morganii] ([WP_193829640.1](https://www.ncbi.nlm.nih.gov/protein/WP_193829640.1?report=genbank&log$=protalign&blast_rank=2&RID=ZXPXDP7A013)) | 99/99(409) | Teichoic acid poly(glycerol phosphate) polymerase transaminase |
| 5 | *tarF_2* | 4982..6154 | 27.87 | CDP-glycerol glycerophosphotransferase family protein [Morganella morganii]  ([WP_126324187.1](https://www.ncbi.nlm.nih.gov/protein/WP_126324187.1?report=genbank&log$=protalign&blast_rank=1&RID=ZXR11FBX013)) | 100/100(390) | Teichoic acid glycerol-phosphate transferase |
| 6 | *arnB* | 6156..7304 | 38.64 | DegT/DnrJ/EryC1/StrS family aminotransferase [Morganella morganii] ([WP_126324188.1](https://www.ncbi.nlm.nih.gov/protein/WP_126324188.1?report=genbank&log$=protalign&blast_rank=1&RID=ZXR4T27G013)) | 100/100(382) | UDP-4-amino-4-deoxy-L-arabinose--oxoglutarate aminotransferase |
| 7 | *GT1* | 7311..7916 | 34.32 | sugar transferase [Arsenophonus endosymbiont of Nilaparvata lugens] (WP_032116130.1) | 69/83(201) | sugar transferase |
| 8 | *pglF* | 8151..9791 | 35.22 | dTDP-glucose-4,6-dehydratase/UDP-glucose 4-epimerase (WeeK) (WbpM) [Xenorhabdus nematophila ATCC 19061] (CBJ88226.1) | 72/84(546) | dTDP-glucose-4,6-dehydratase/UDP-glucose 4-epimerase |
| 9 | *trmL* | 9797..10300 | 51.58 | tRNA(uridine(34)/cytosine(34)/5-carboxymethylaminomethyluridine(34)-2'-O)-methyltransferase TrmL [Morganella morganii]  ([WP_025152359.1](https://www.ncbi.nlm.nih.gov/protein/WP_025152359.1?report=genbank&log$=protalign&blast_rank=2&RID=ZXRGH2N201R)) | 99/100(167) | tRNA(cytidine(34)-2'-O)-methyltransferase |
| 10 | *cysE* | complement(10374..11174) | 55.55 | serine O-acetyltransferase [Morganella morganii] ([WP_004236641.1](https://www.ncbi.nlm.nih.gov/protein/WP_004236641.1?report=genbank&log$=protalign&blast_rank=3&RID=ZXRPH6UW013)) | 99/100(266) | Serine acetyltransferase |
| 11 | *gpsA* | complement(11209..12222) | 57.49 | NAD(P)H-dependent glycerol-3-phosphate dehydrogenase [Morganella morganii]  ([WP_163654164.1](https://www.ncbi.nlm.nih.gov/protein/WP_163654164.1?report=genbank&log$=protalign&blast_rank=2&RID=ZXRVYYUC016)) | 99/100(337) | Glycerol-3-phosphate dehydrogenase [NAD(P)+] |

G6345 (type 4)

| Orf no. | Gene name | Position of gene | G+C content(%) | Similar protein(s), strain(s) (Genbank accession No.) | %Identical/%Similar (total No. of aa) | Putative function of protein |
| --- | --- | --- | --- | --- | --- | --- |
| 1 | *orf1* | 1..897 | 29.76 | phosphotransferase [Morganella morganii]   ([WP_087825451.1](https://www.ncbi.nlm.nih.gov/protein/WP_087825451.1?report=genbank&log$=protalign&blast_rank=1&RID=009GSHZ601R)) | 99/99（298） | hypothetical protein |
| 2 | *orf2* | 881..1816 | 35.89 | DMT family transporter [Morganella morganii]  ([WP_124537815.1](https://www.ncbi.nlm.nih.gov/protein/WP_124537815.1?report=genbank&log$=protalign&blast_rank=1&RID=009M3C9601R)) | 100/100(311) | hypothetical protein |
| 3 | *orf3* | 1813..2505 | 32.17 | NTP transferase domain-containing protein [Morganella morganii] ([WP_049247114.1](https://www.ncbi.nlm.nih.gov/protein/WP_049247114.1?report=genbank&log$=protalign&blast_rank=1&RID=009PWM6301R)) | 96/98(230) | NTP transferase domain-containing protein |
| 4 | *wzx* | 2492..3760 | 26.31 | oligosaccharide flippase family protein [Salmonella enterica subsp. enterica serovar Kiambu] ([EEE0989068.1](https://www.ncbi.nlm.nih.gov/protein/EEE0989068.1?report=genbank&log$=protalign&blast_rank=1&RID=009U9GBM01R)) | 71/85(422) | flippase |
| 5 | *orf5* | 3745..4455 | 25.31 | LicD family protein [Xenorhabdus mauleonii]  [WP_092512116.1](https://www.ncbi.nlm.nih.gov/protein/WP_092512116.1?report=genbank&log$=protalign&blast_rank=1&RID=009XJ85B013) | 43/67(236) | hypothetical protein |
| 6 | *wzy* | 4457..5641 | 25.06 | EpsG family protein [Xenorhabdus mauleonii]   ([WP_092512114.1](https://www.ncbi.nlm.nih.gov/protein/WP_092512114.1?report=genbank&log$=protalign&blast_rank=1&RID=00A1G46T016)) | 47/64(394) | polymerase |
| 7 | *GT1* | 5654..6676 | 26.78 | WfgC [Haemophilus parainfluenzae] (AGO01083.1) | 53/66(340) | Glycosyl transferase |
| 8 | *GT2* | 6678..7418 | 27.8 | glycosyltransferase family 2 protein [Morganella morganii]  ([WP_153643057.1](https://www.ncbi.nlm.nih.gov/protein/WP_153643057.1?report=genbank&log$=protalign&blast_rank=1&RID=00A8UT2301R)) | 90/93(246) | UDP-Glc:alpha-D-GlcNAc-diphosphoundecaprenol beta-1,3-glucosyltransferase WfgD |
| 9 | *gnu* | 7420..8370 | 40.37 | NAD-dependent epimerase/dehydratase family protein [Morganella morganii] ([WP_153643058.1](https://www.ncbi.nlm.nih.gov/protein/WP_153643058.1?report=genbank&log$=protalign&blast_rank=1&RID=00ABX5S001R)) | 99/99(316) | N-acetyl-alpha-D-glucosaminyl-diphospho-ditrans, octacis-undecaprenol 4-epimerase |
| 10 | *trmL* | 8426..8929 | 49.8 | tRNA(uridine(34)/cytosine(34)/5-carboxymethylaminomethyluridine(34)-2'-O)-methyltransferase TrmL [Morganella morganii]   ([WP_025152359.1](https://www.ncbi.nlm.nih.gov/protein/WP_025152359.1?report=genbank&log$=protalign&blast_rank=2&RID=00AEVXV801R)) | 99/100(167) | tRNA(cytidine(34)-2'-O)-methyltransferase |
| 11 | *cysE* | complement(9003..9803) | 55.05 | serine O-acetyltransferase [Morganella morganii]  ([HAT1527377.1](https://www.ncbi.nlm.nih.gov/protein/HAT1527377.1?report=genbank&log$=protalign&blast_rank=1&RID=00AHXFN301R)) | 100/100(266) | Serine acetyltransferase |
| 12 | *gpsA* | complement(9837..10850) | 58.08 | NAD(P)H-dependent glycerol-3-phosphate dehydrogenase [Morganella morganii] ([WP_163654164.1](https://www.ncbi.nlm.nih.gov/protein/WP_163654164.1?report=genbank&log$=protalign&blast_rank=2&RID=00AN8MMZ01R)) | 99/100(337) | Glycerol-3-phosphate dehydrogenase [NAD(P)+] |

G6352 (type 5)

| Orf no. | Gene name | Position of gene | G+C content(%) | Similar protein(s), strain(s) (Genbank accession No.) | %Identical/%Similar (total No. of aa) | Putative function of protein |
| --- | --- | --- | --- | --- | --- | --- |
| 1 | *orf1* | 1..897 | 30.21 | phosphotransferase [Morganella morganii] ([WP_165894764.1](https://www.ncbi.nlm.nih.gov/protein/WP_165894764.1?report=genbank&log$=protalign&blast_rank=1&RID=ZZMM8E01016)) | 100/100（298） | hypothetical protein |
| 2 | *orf2* | 881..1816 | 35.47 | DMT family transporter [Morganella morganii] ([WP_036425138.1](https://www.ncbi.nlm.nih.gov/protein/WP_036425138.1?report=genbank&log$=protalign&blast_rank=2&RID=ZZMRABEP016)) | 99/100（311） | hypothetical protein |
| 3 | *orf3* | 1813..2499 | 32.16 | NTP transferase domain-containing protein [Morganella morganii]([WP_087825452.1](https://www.ncbi.nlm.nih.gov/protein/WP_087825452.1?report=genbank&log$=protalign&blast_rank=2&RID=ZZMV1EP7016)) | 99/100（228） | UTP--glucose-1-phosphate uridylyltransferase |
| 4 | *tarF* | 2514..3689 | 29.25 | CDP-glycerol glycerophosphotransferase family protein [Morganella morganii] ([WP_101924445.1](https://www.ncbi.nlm.nih.gov/protein/WP_101924445.1?report=genbank&log$=protalign&blast_rank=2&RID=ZZMYNBZJ016)) | 99/100（391） | Teichoic acid poly(glycerol phosphate) polymerase |
| 5 | *GT1* | 3691..4779 | 31.22 | glycosyltransferase family 4 protein [Salmonella enterica subsp. enterica serovar Newport] (EBX5144967.1) | 99/99（362） | glycosyltransferase family 4 protein |
| 6 | *wzy* | 4769..5845 | 28.22 | EpsG family protein [Xenorhabdus szentirmaii] (WP_038239940.1) | 47/66（358） | polymerase |
| 7 | *tagD* | 5855..6325 | 28.23 | glycerol-3-phosphate cytidylyltransferase [Morganella morganii] ([WP_087696818.1](https://www.ncbi.nlm.nih.gov/protein/WP_087696818.1?report=genbank&log$=protalign&blast_rank=1&RID=ZZNB79SV01R)) | 100/100（156） | Glycerol-3-phosphate cytidylyltransferase |
| 8 | *orf8* | 6325..7326 | 29.04 | sulfotransferase [Morganella morganii] ([WP_165894765.1](https://www.ncbi.nlm.nih.gov/protein/WP_165894765.1?report=genbank&log$=protalign&blast_rank=3&RID=ZZNDZPB201R)) | 99/100（333） | hypothetical protein |
| 9 | *GT2* | 7352..8161 | 30.24 | glycosyltransferase [Morganella morganii] ([WP_087825454.1](https://www.ncbi.nlm.nih.gov/protein/WP_087825454.1?report=genbank&log$=protalign&blast_rank=2&RID=ZZNHZHPR01R)) | 96/98（269） | UDP-Gal:alpha-D-GlcNAc-diphosphoundecaprenol beta-1,3-galactosyltransferase |
| 10 | *orf10* | 8166..8441 | 29.71 | hypothetical protein [Morganella morganii] (WP_115072348.1) | 99/99（91） | hypothetical protein |
| 11 | *orf11* | 8497..8895 | 30.32 | hypothetical protein [Morganella morganii] (WP_155251734.1) | 99/99(132) | hypothetical protein |
| 12 | *wzx* | 8892..10340 | 26.84 | oligosaccharide flippase family protein [Arsenophonus nasoniae](WP_026821942.1) | 99/99（482） | flippase |
| 13 | *gnu* | 10358..11308 | 40.9 | NAD-dependent epimerase/dehydratase family protein [Morganella morganii] ([WP_062773462.1](https://www.ncbi.nlm.nih.gov/protein/WP_062773462.1?report=genbank&log$=protalign&blast_rank=1&RID=ZZNWFA8M01R)) | 100/100（316） | N-acetyl-alpha-D-glucosaminyl-diphospho-ditrans, octacis-undecaprenol 4-epimerase |
| 14 | *trmL* | 11364..11867 | 51.38 | tRNA(uridine(34)/cytosine(34)/5-carboxymethylaminomethyluridine(34)-2'-O)-methyltransferase TrmL [Morganella morganii]   ([WP_025152359.1](https://www.ncbi.nlm.nih.gov/protein/WP_025152359.1?report=genbank&log$=protalign&blast_rank=2&RID=ZZNZGEKC016)) | 99/100（167） | tRNA (cytidine(34)-2'-O)-methyltransferase |
| 15 | *cysE* | complement(11941..12741) | 55.18 | serine O-acetyltransferase [Morganella morganii]   ([WP_064483679.1](https://www.ncbi.nlm.nih.gov/protein/WP_064483679.1?report=genbank&log$=protalign&blast_rank=2&RID=ZZP2RAR3016)) | 100/100（267） | Serine acetyltransferase |
| 16 | *gpsA* | complement(12776..13789) | 57.1 | NAD(P)H-dependent glycerol-3-phosphate dehydrogenase [Morganella morganii] ([WP_163654164.1](https://www.ncbi.nlm.nih.gov/protein/WP_163654164.1?report=genbank&log$=protalign&blast_rank=2&RID=ZZP715B3013)) | 99/100（337） | Glycerol-3-phosphate dehydrogenase [NAD(P)+] |

G6354 (type 6)

| Orf no. | Gene name | Position of gene | G+C content(%) | Similar protein(s), strain(s) (Genbank accession No.) | %Identical/%Similar (total No. of aa) | Putative function of protein |
| --- | --- | --- | --- | --- | --- | --- |
| 1 | *wzx* | 1..1368 | 28.43 | oligosaccharide flippase family protein [Escherichia coli]  (WP_095764318.1) | 45/67(455) | flippase |
| 2 | *orf2* | 1371..2717 | 29.69 | Coenzyme F420 hydrogenase/dehydrogenase, beta subunit C-terminal domain [Morganella morganii]   ([WP_123569632.1](https://www.ncbi.nlm.nih.gov/protein/WP_123569632.1?report=genbank&log$=protalign&blast_rank=1&RID=ZZ35KJ6G016)) | 100/100(448) | hypothetical protein |
| 3 | *orf3* | 2741..3868 | 28.19 | polysaccharide pyruvyl transferase family protein [Morganella morganii] ([WP_123569631.1](https://www.ncbi.nlm.nih.gov/protein/WP_123569631.1?report=genbank&log$=protalign&blast_rank=1&RID=ZZ39TNXD013)) | 100/100(375) | polysaccharide pyruvyl transferase family  protein |
| 4 | *wzy* | 3917..5182 | 26.14 | oligosaccharide repeat unit polymerase [Morganella morganii]  ([WP_123569630.1](https://www.ncbi.nlm.nih.gov/protein/WP_123569630.1?report=genbank&log$=protalign&blast_rank=1&RID=ZZ3DABCE016)) | 100/100(421) | hypothetical protein |
| 5 | *GT1* | 5179..5973 | 27.29 | glycosyltransferase [Morganella morganii]   ([WP_123569629.1](https://www.ncbi.nlm.nih.gov/protein/WP_123569629.1?report=genbank&log$=protalign&blast_rank=1&RID=ZZ3GXF7N016)) | 99/100(264) | glycosyltransferase family 2 protein |
| 6 | *ugd* | 5983..7149 | 30.59 | nucleotide sugar dehydrogenase [Morganella morganii]  ([WP_186749557.1](https://www.ncbi.nlm.nih.gov/protein/WP_186749557.1?report=genbank&log$=protalign&blast_rank=2&RID=ZZKFMWDN01R)) | 99/99(388) | UDP-glucose 6-dehydrogenase |
| 7 | *GT2* | 7142..7921 | 29.65 | glycosyltransferase [Morganella morganii] ([WP_158604696.1](https://www.ncbi.nlm.nih.gov/protein/WP_158604696.1?report=genbank&log$=protalign&blast_rank=1&RID=ZZKK8MX901R)) | 100/100（259） | Putative teichuronic acid biosynthesis glycosyltransferase TuaG |
| 8 | *arnB* | 7921..9075 | 39.3 | DegT/DnrJ/EryC1/StrS family aminotransferase [Morganella morganii] ([WP_123569626.1](https://www.ncbi.nlm.nih.gov/protein/WP_123569626.1?report=genbank&log$=protalign&blast_rank=1&RID=ZZKPAHAK01R)) | 100/100（384） | UDP-4-amino-4-deoxy-L-arabinose--oxoglutarate aminotransferase |
| 9 | *GT3* | 9082..9687 | 34.72 | sugar transferase [Morganella morganii]([WP_186749569.1](https://www.ncbi.nlm.nih.gov/protein/WP_186749569.1?report=genbank&log$=protalign&blast_rank=2&RID=ZZKUGA6U013)) | 99/100（197） | UDP-glucose:undecaprenyl-phosphate glucose-1-phosphate transferase |
| 10 | *pglF* | 9700..11562 | 34.51 | polysaccharide biosynthesis protein [Morganella morganii]  ([WP_123569625.1](https://www.ncbi.nlm.nih.gov/protein/WP_123569625.1?report=genbank&log$=protalign&blast_rank=1&RID=ZZKYN88F016)) | 100/100（620） | UDP-N-acetyl-alpha-D-glucosamine C6 dehydratase |
| 11 | *trmL* | 11568..12071 | 49.6 | tRNA(uridine(34)/cytosine(34)/5-carboxymethylaminomethyluridine(34)-2'-O)-methyltransferase TrmL [Morganella morganii]  ([WP_072871530.1](https://www.ncbi.nlm.nih.gov/protein/WP_072871530.1?report=genbank&log$=protalign&blast_rank=2&RID=ZZM1W092016)) | 99/100（167） | tRNA(cytidine(34)-2'-O)-methyltransferase |
| 12 | *cysE* | complement(12145..12945) | 56.05 | serine O-acetyltransferase [Morganella morganii]  ([WP_004236641.1](https://www.ncbi.nlm.nih.gov/protein/WP_004236641.1?report=genbank&log$=protalign&blast_rank=3&RID=ZZM5CBTS016)) | 99/100（266） | Serine acetyltransferase |
| 13 | *gpsA* | complement(12979..13992) | 57.1 | NAD(P)H-dependent glycerol-3-phosphate dehydrogenase [Morganella morganii]([WP_163654164.1](https://www.ncbi.nlm.nih.gov/protein/WP_163654164.1?report=genbank&log$=protalign&blast_rank=2&RID=ZZM9AAM4013)) | 99/100（337） | Glycerol-3-phosphate dehydrogenase [NAD(P)+] |

G6359 (type 7)

| Orf no. | Gene name | Position of gene | G+C content(%) | Similar protein(s), strain(s) (Genbank accession No.) | %Identical/%Similar (total No. of aa) | Putative function of protein |
| --- | --- | --- | --- | --- | --- | --- |
| 1 | *rmlA* | 1..873 | 40.32 | glucose-1-phosphate thymidylyltransferase [Morganella morganii] ([KJF78563.1](https://www.ncbi.nlm.nih.gov/protein/KJF78563.1?report=genbank&log$=protalign&blast_rank=1&RID=ZXU3TZT601R)) | 99/100(290) | Glucose-1-phosphate thymidylyltransferase 2 |
| 2 | *fdtA* | 870..1265 | 34.59 | FdtA/QdtA family cupin domain-containing protein [Morganella morganii] ([WP_152696941.1](https://www.ncbi.nlm.nih.gov/protein/WP_152696941.1?report=genbank&log$=protalign&blast_rank=1&RID=ZXU7R38R01R)) | 98/98(131) | TDP-4-oxo-6-deoxy-alpha-D-glucose-3, 4-oxoisomerase |
| 3 | *fdtC* | 1284..1805 | 30.07 | GNAT family N-acetyltransferase [Morganella morganii]  ([WP_152696940.1](https://www.ncbi.nlm.nih.gov/protein/WP_152696940.1?report=genbank&log$=protalign&blast_rank=1&RID=ZXUB810A01R)) | 99/100(173) | hypothetical protein |
| 4 | *fdtB* | 1836..2957 | 34.04 | DegT/DnrJ/EryC1/StrS family aminotransferase [Morganella morganii] ([WP_152696939.1](https://www.ncbi.nlm.nih.gov/protein/WP_152696939.1?report=genbank&log$=protalign&blast_rank=1&RID=ZXUEYPHN016)) | 95/96(372) | dTDP-3-amino-3,6-dideoxy-alpha-D-galactopyranose transaminase |
| 5 | *GT1* | 2969..3850 | 29.25 | glycosyltransferase [Morganella morganii]([WP_127377996.1](https://www.ncbi.nlm.nih.gov/protein/WP_127377996.1?report=genbank&log$=protalign&blast_rank=1&RID=ZXUKA5FU013)) | 94/96(293) | putative glycosyltransferase EpsJ |
| 6 | *wzx* | 3868..5124 | 30.07 | O-antigen translocase [Morganella morganii]([WP_127377997.1](https://www.ncbi.nlm.nih.gov/protein/WP_127377997.1?report=genbank&log$=protalign&blast_rank=1&RID=ZXUSDMYX01R)) | 97/98(418) | Lipid III flippase |
| 7 | *GT2* | 5097..6107 | 26.74 | glycosyltransferase family 2 protein [Morganella morganii]  ([WP_127377998.1](https://www.ncbi.nlm.nih.gov/protein/WP_127377998.1?report=genbank&log$=protalign&blast_rank=1&RID=ZXUW9THM01R)) | 94/97(316) | putative glycosyltransferase EpsJ |
| 8 | *wzy* | 6101..7234 | 26.54 | EpsG family protein [Morganella morganii]([WP_127377999.1](https://www.ncbi.nlm.nih.gov/protein/WP_127377999.1?report=genbank&log$=protalign&blast_rank=1&RID=ZXUYNY3H01R)) | 94/96(372) | hypothetical protein |
| 9 | *GT3* | 7231..8334 | 36.04 | glycosyltransferase [Morganella morganii]([WP_127378000.1](https://www.ncbi.nlm.nih.gov/protein/WP_127378000.1?report=genbank&log$=protalign&blast_rank=1&RID=ZXV21T9V01R)) | 83/88(363) | N-acetyl-alpha-D-glucosaminyl L-malate synthase |
| 10 | *GT4* | 8331..9368 | 31.69 | glycosyltransferase family 1 protein [Vibrio vulnificus]  ([HAS8351703.1](https://www.ncbi.nlm.nih.gov/protein/HAS8351703.1?report=genbank&log$=protalign&blast_rank=1&RID=ZXV5JM4T013)) | 95/97(345) | O-antigen biosynthesis glycosyltransferase WbnH |
| 11 | *gnu* | 9375..10325 | 40.16 | NAD-dependent epimerase/dehydratase family protein [Salmonella enterica subsp. enterica serovar Virchow]   ([EBN0073238.1](https://www.ncbi.nlm.nih.gov/protein/EBN0073238.1?report=genbank&log$=protalign&blast_rank=2&RID=ZXVA3BCE016)) | 99/100(316) | N-acetyl-alpha-D-glucosaminyl-diphospho-ditrans, octacis-undecaprenol 4-epimerase |
| 12 | *trmL* | 10381..10884 | 49.8 | tRNA(uridine(34)/cytosine(34)/5-carboxymethylaminomethyluridine(34)-2'-O)-methyltransferase TrmL [Morganella morganii]   ([WP_025152359.1](https://www.ncbi.nlm.nih.gov/protein/WP_025152359.1?report=genbank&log$=protalign&blast_rank=1&RID=ZXVER98N01R)) | 100/100(167) | tRNA(cytidine(34)-2'-O)-methyltransferase |
| 13 | *cysE* | complement(10958..11758) | 54.3 | serine O-acetyltransferase [Morganella morganii]  ([WP_004236641.1](https://www.ncbi.nlm.nih.gov/protein/WP_004236641.1?report=genbank&log$=protalign&blast_rank=2&RID=ZXVJ1FKA01R)) | 99/100(266) | Serine acetyltransferase |
| 14 | *gpsA* | complement(11792..12805) | 57.69 | NAD(P)H-dependent glycerol-3-phosphate dehydrogenase [Morganella morganii] ([WP_163654164.1](https://www.ncbi.nlm.nih.gov/protein/WP_163654164.1?report=genbank&log$=protalign&blast_rank=2&RID=ZXVNEBUU01R)) | 99/100(337) | Glycerol-3-phosphate dehydrogenase [NAD(P)+] |

G6360 (type 8)

| Orf no. | Gene name | Position of gene | G+C content(%) | Similar protein(s), strain(s) (Genbank accession No.) | %Identical/%Similar (total No. of aa) | Putative function of protein |
| --- | --- | --- | --- | --- | --- | --- |
| 1 | *orf1* | 1..897 | 30.21 | phosphotransferase [Morganella morganii][WP_165894764.1](https://www.ncbi.nlm.nih.gov/protein/WP_165894764.1?report=genbank&log$=protalign&blast_rank=2&RID=006XYKPU016) | 99/100（298） | phosphotransferase |
| 2 | *orf2* | 881..1816 | 35.47 | DMT family transporter [Morganella morganii] ([WP_036425138.1](https://www.ncbi.nlm.nih.gov/protein/WP_036425138.1?report=genbank&log$=protalign&blast_rank=2&RID=0073ZXNC016)) | 99/100(311) | DMT family transporter |
| 3 | *orf3* | 1813..2499 | 32.16 | NTP transferase domain-containing protein [Morganella morganii]   ([WP_087825452.1](https://www.ncbi.nlm.nih.gov/protein/WP_087825452.1?report=genbank&log$=protalign&blast_rank=2&RID=0079FCDY01R)) | 99/100(228) | UTP--glucose-1-phosphate uridylyltransferase |
| 4 | *tarF* | 2514..3689 | 29.33 | MULTISPECIES: CDP-glycerol glycerophosphotransferase family protein [Morganella]  ([WP_079549407.1](https://www.ncbi.nlm.nih.gov/protein/WP_079549407.1?report=genbank&log$=protalign&blast_rank=1&RID=007GN61H016)) | 100/100(391) | CDP-glycerol glycerophosphotransferase family protein" |
| 5 | *GT1* | 3691..4779 | 31.31 | glycosyltransferase [Morganella morganii] ([WP_062773242.1](https://www.ncbi.nlm.nih.gov/protein/WP_062773242.1?report=genbank&log$=protalign&blast_rank=1&RID=007NP4Z901R)) | 99/99(362) | N, N'-diacetylbacillosaminyl-diphospho-undecaprenol alpha-1,3-N-acetylgalactosaminyltransferase |
| 6 | *wzy* | 4769..5845 | 28.22 | EpsG family protein [Morganella morganii]  ([WP_182095897.1](https://www.ncbi.nlm.nih.gov/protein/WP_182095897.1?report=genbank&log$=protalign&blast_rank=1&RID=007TDXDY013)) | 99/100(358) | polymerase |
| 7 | *tagD* | 5855..6325 | 28.23 | glycerol-3-phosphate cytidylyltransferase [Morganella morganii]([WP_087696818.1](https://www.ncbi.nlm.nih.gov/protein/WP_087696818.1?report=genbank&log$=protalign&blast_rank=1&RID=008053W7013)) | 100/100(156) | Glycerol-3-phosphate cytidylyltransferase |
| 8 | *orf8* | 6421..7326 | 27.92 | MULTISPECIES: sulfotransferase family protein [Morganella] ([WP_036417427.1](https://www.ncbi.nlm.nih.gov/protein/WP_036417427.1?report=genbank&log$=protalign&blast_rank=1&RID=0085CT8U016)) | 100/100(333) | sulfotransferase |
| 9 | *GT2* | 7352..8161 | 30.24 | glycosyltransferase [Morganella morganii] ([WP_087825454.1](https://www.ncbi.nlm.nih.gov/protein/WP_087825454.1?report=genbank&log$=protalign&blast_rank=2&RID=008CM0K8016)) | 96/98(269) | UDP-Gal:alpha-D-GlcNAc-diphosphoundecaprenol beta-1,3-galactosyltransferase |
| 10 | *orf10* | 8166..9314 | 27.5 | hypothetical protein [Morganella morganii]   ([WP_079549459.1](https://www.ncbi.nlm.nih.gov/protein/WP_079549459.1?report=genbank&log$=protalign&blast_rank=1&RID=008KET2H016)) | 100/100(382) | hypothetical protein |
| 11 | *wzx* | 9311..10759 | 27.12 | oligosaccharide flippase family protein [Morganella morganii]  ([WP_036425127.1](https://www.ncbi.nlm.nih.gov/protein/WP_036425127.1?report=genbank&log$=protalign&blast_rank=1&RID=008RTK4B01R)) | 100/100(482) | Putative O-antigen transporter |
| 12 | *gnu* | 10777..11727 | 41.21 | NAD-dependent epimerase/dehydratase family protein [Morganella morganii]   ([WP_062773462.1](https://www.ncbi.nlm.nih.gov/protein/WP_062773462.1?report=genbank&log$=protalign&blast_rank=2&RID=008W2FDD013)) | 99/99(316) | N-acetyl-alpha-D-glucosaminyl-diphospho-ditrans, octacis-undecaprenol 4-epimerase |
| 13 | *trmL* | 11783..12286 | 51.58 | tRNA(uridine(34)/cytosine(34)/5-carboxymethylaminomethyluridine(34)-2'-O)-methyltransferase TrmL [Morganella morganii]  ([WP_025152359.1](https://www.ncbi.nlm.nih.gov/protein/WP_025152359.1?report=genbank&log$=protalign&blast_rank=2&RID=0090362H013)) | 99/100(167) | tRNA (cytidine(34)-2'-O)-methyltransferase |
| 14 | *cysE* | complement(12360..13160) | 55.68 | serine O-acetyltransferase [Morganella morganii]  ([WP_064483679.1](https://www.ncbi.nlm.nih.gov/protein/WP_064483679.1?report=genbank&log$=protalign&blast_rank=2&RID=00954M2G013)) | 100/100(267) | Serine acetyltransferase |
| 15 | *gpsA* | complement(13195..14208) | 56.7 | NAD(P)H-dependent glycerol-3-phosphate dehydrogenase [Morganella morganii] ([WP_049247126.1](https://www.ncbi.nlm.nih.gov/protein/WP_049247126.1?report=genbank&log$=protalign&blast_rank=1&RID=009A07V5013)) | 100/100(337) | Glycerol-3-phosphate dehydrogenase [NAD(P)+] |

G6364 (type 9)

| Orf no. | Gene name | Position of gene | G+C content(%) | Similar protein(s), strain(s) (Genbank accession No.) | %Identical/%Similar (total No. of aa) | Putative function of protein |
| --- | --- | --- | --- | --- | --- | --- |
| 1 | *rmlD* | 1..900 | 33.0 | dTDP-4-dehydrorhamnose reductase [Morganella morganii] ([WP_112549944.1](https://www.ncbi.nlm.nih.gov/protein/WP_112549944.1?report=genbank&log$=protalign&blast_rank=1&RID=ZXSA1KR7013) ) | 100/100(299) | dTDP-4-dehydrorhamnose reductase |
| 2 | *rmlA* | 904..1776 | 36.31 | glucose-1-phosphate thymidylyltransferase RfbA [Morganella morganii] ([WP_186849510.1](https://www.ncbi.nlm.nih.gov/protein/WP_186849510.1?report=genbank&log$=protalign&blast_rank=2&RID=ZXSEDCRZ016)) | 99/100(290) | Glucose-1-phosphate thymidylyltransferase 1 |
| 3 | *rmlC* | 1778..2320 | 32.96 | dTDP-4-dehydrorhamnose 3,5-epimerase [Morganella morganii] ([WP_186849509.1](https://www.ncbi.nlm.nih.gov/protein/WP_186849509.1?report=genbank&log$=protalign&blast_rank=1&RID=ZXSK6Y0V016)) | 96/98(180) | dTDP-4-dehydrorhamnose 3,5-epimerase |
| 4 | *GT1* | 2317..3075 | 26.87 | glycosyltransferase family 2 protein [Morganella morganii]  ([WP_186849508.1](https://www.ncbi.nlm.nih.gov/protein/WP_186849508.1?report=genbank&log$=protalign&blast_rank=1&RID=ZXSPK6J201R)) | 99/99(254) | Rhamnosyltransferase WbbL |
| 5 | *wzx* | 3079..4302 | 28.83 | flippase [Morganella morganii] ([WP_112550562.1](https://www.ncbi.nlm.nih.gov/protein/WP_112550562.1?report=genbank&log$=protalign&blast_rank=1&RID=ZXSW84D501R)) | 100/100(407) | Putative O-antigen transporter |
| 6 | *glf* | 4313..5413 | 28.88 | UDP-galactopyranose mutase [Morganella morganii]  ([WP_112550561.1](https://www.ncbi.nlm.nih.gov/protein/WP_112550561.1?report=genbank&log$=protalign&blast_rank=1&RID=ZXT034W901R) ) | 100/100(366) | UDP-galactopyranose mutase |
| 7 | *wzy* | 5407..6513 | 26.64 | hypothetical protein [Morganella morganii]  ([WP_112550560.1](https://www.ncbi.nlm.nih.gov/protein/WP_112550560.1?report=genbank&log$=protalign&blast_rank=1&RID=ZXT47VUK01R)) | 100/100(368) | hypothetical protein |
| 8 | *GT2* | 6479..7276 | 27.44 | glycosyltransferase [Morganella morganii]([WP_112550559.1](https://www.ncbi.nlm.nih.gov/protein/WP_112550559.1?report=genbank&log$=protalign&blast_rank=1&RID=ZXT7UG41016)) | 100/100(265) | O-antigen biosynthesis glycosyltransferase WbnJ |
| 9 | *GT3* | 7266..8405 | 27.54 | glycosyltransferase [Morganella morganii]([WP_112550558.1](https://www.ncbi.nlm.nih.gov/protein/WP_112550558.1?report=genbank&log$=protalign&blast_rank=1&RID=ZXTB3JCA01R)) | 100/100(379) | Putative teichuronic acid biosynthesis glycosyltransferase TuaC |
| 10 | *IS* | 8980..9666 | 49.78 | IS3 family transposase [Morganella morganii] ([WP_107680165.1](https://www.ncbi.nlm.nih.gov/protein/WP_107680165.1?report=genbank&log$=protalign&blast_rank=1&RID=ZXTJMXNJ01R)) | 99/99(370) | IS3 family transposase |
| 11 | *trmL* | 9712..10215 | 49.0 | tRNA(uridine(34)/cytosine(34)/5-carboxymethylaminomethyluridine(34)-2'-O)-methyltransferase TrmL [Morganella morganii]   ([WP_025152359.1](https://www.ncbi.nlm.nih.gov/protein/WP_025152359.1?report=genbank&log$=protalign&blast_rank=1&RID=ZXTPJ4TF01R)) | 100/100(167) | tRNA (cytidine(34)-2'-O)-methyltransferase |
| 12 | *cysE* | complement(10289..11089) | 54.05 | serine O-acetyltransferase [Morganella morganii] ([WP_112550557.1](https://www.ncbi.nlm.nih.gov/protein/WP_112550557.1?report=genbank&log$=protalign&blast_rank=1&RID=ZXTU3TJ401R)) | 100/100(266) | Serine acetyltransferase |
| 13 | *gpsA* | complement(11123..12136) | 58.08 | NAD(P)H-dependent glycerol-3-phosphate dehydrogenase [Morganella morganii] ([WP_025152358.1](https://www.ncbi.nlm.nih.gov/protein/WP_025152358.1?report=genbank&log$=protalign&blast_rank=1&RID=ZXTY3UX901R)) | 100/100(337) | Glycerol-3-phosphate dehydrogenase [NAD(P)+] |

G6367 (type 10)

| Orf no. | Gene name | Position of gene | G+C content(%) | Similar protein(s), strain(s) (Genbank accession No.) | %Identical/%Similar (total No. of aa) | Putative function of protein |
| --- | --- | --- | --- | --- | --- | --- |
| 1 | *rmlA* | 1..873 | 39.63 | glucose-1-phosphate thymidylyltransferase RfbA [Morganella morganii] ( [WP_152713705.1](https://www.ncbi.nlm.nih.gov/protein/WP_152713705.1?report=genbank&log$=protalign&blast_rank=1&RID=ZWTCUYE4013)) | 99/100(290) | Glucose-1-phosphate thymidylyltransferase 2 |
| 2 | *fdtA* | 870..1277 | 32.84 | dTDP-6-deoxy-3,4-keto-hexulose isomerase [Morganella morganii] ( [KJF78565.1](https://www.ncbi.nlm.nih.gov/protein/KJF78565.1?report=genbank&log$=protalign&blast_rank=2&RID=ZWU97CUD013)) | 99/99(135) | TDP-4-oxo-6-deoxy-alpha-D-glucose-3,4-oxoisomerase |
| 3 | *fdtC* | 1240..1821 | 29.2 | GNAT family N-acetyltransferase [Morganella morganii] ( [WP_195432641.1](https://www.ncbi.nlm.nih.gov/protein/WP_195432641.1?report=genbank&log$=protalign&blast_rank=1&RID=ZWUMYF2K013)) | 98/98(175) | acetyltransferase |
| 4 | *fdtB* | 1838..2956 | 34.67 | GNAT family N-acetyltransferase [Morganella morganii] ( [WP_195432641.1](https://www.ncbi.nlm.nih.gov/protein/WP_195432641.1?report=genbank&log$=protalign&blast_rank=1&RID=ZWUMYF2K013)) | 98/98(175) | dTDP-3-amino-3,6-dideoxy-alpha-D-galactopyranose transaminase |
| 5 | *wzx* | 2961..4238 | 26.99 | Polysaccharide biosynthesis protein [Providencia rettgeri] ([APC10621.1](https://www.ncbi.nlm.nih.gov/protein/APC10621.1?report=genbank&log$=protalign&blast_rank=1&RID=ZWV318NK013)) | 67/81(419) | Lipid III flippase |
| 6 | *orf6* | 4216..4845 | 21.9 | haloacid dehalogenase-like hydrolase [Providencia alcalifaciens] ( [WP_006663205.1](https://www.ncbi.nlm.nih.gov/protein/WP_006663205.1?report=genbank&log$=protalign&blast_rank=1&RID=ZWV99WM9013)) | 46/62(212) | hypothetical protein |
| 7 | *GT1* | 4926..5828 | 26.35 | glycosyltransferase family 2 protein [Escherichia coli] (  [WP_095530515.1](https://www.ncbi.nlm.nih.gov/protein/WP_095530515.1?report=genbank&log$=protalign&blast_rank=1&RID=ZWVE7HFS013)) | 48/65(300) | Teichoic acid poly(glycerol phosphate) polymerase |
| 8 | *wzy* | 5818..7125 | 26.6 | oligosaccharide repeat unit polymerase [Providencia rettgeri] ( [WP_110731542.1](https://www.ncbi.nlm.nih.gov/protein/WP_110731542.1?report=genbank&log$=protalign&blast_rank=1&RID=ZWVKY6KW016)) | 48/64(436) | polymerase |
| 9 | *orf9* | 7276..8067 | 26.38 | hypothetical protein B7P19_14550 [Enterobacter sp. Crenshaw]( [AUM04363.1](https://www.ncbi.nlm.nih.gov/protein/AUM04363.1?report=genbank&log$=protalign&blast_rank=1&RID=ZWVT6JJR016)) | 47/63(262) | hypothetical protein |
| 10 | *GT2* | 8075..8899 | 32.72 | glycosyltransferase [Morganella morganii] ( [WP_195432642.1](https://www.ncbi.nlm.nih.gov/protein/WP_195432642.1?report=genbank&log$=protalign&blast_rank=1&RID=ZWW2F68801R)) | 99/99(268) | UDP-Gal:alpha-D-GlcNAc-diphosphoundecaprenol beta-1,3-galactosyltransferase |
| 11 | *gnu* | 8909..9859 | 39.85 | NAD-dependent epimerase/dehydratase family protein [Morganella morganii] ( [WP_153643058.1](https://www.ncbi.nlm.nih.gov/protein/WP_153643058.1?report=genbank&log$=protalign&blast_rank=1&RID=ZWW6NRJH01R)) | 99/99(316) | N-acetyl-alpha-D-glucosaminyl-diphospho-ditrans, octacis-undecaprenol 4-epimerase |
| 12 | *trmL* | 9915..10418 | 49.2 | tRNA (uridine(34)/cytosine(34)/5-carboxymethylaminomethyluridine(34)-2'-O)-methyltransferase TrmL [Morganella morganii] ( [WP_123569624.1](https://www.ncbi.nlm.nih.gov/protein/WP_123569624.1?report=genbank&log$=protalign&blast_rank=1&RID=ZX2JM2PG013)) | 99/99(167) | tRNA (cytidine(34)-2'-O)-methyltransferase |
| 13 | *eptC* | complement(10482..12017) | 28.32 | sulfatase-like hydrolase/transferase [Morganella morganii]( [WP_195432596.1](https://www.ncbi.nlm.nih.gov/protein/WP_195432596.1?report=genbank&log$=protalign&blast_rank=1&RID=ZX2VXDKV013) ) | 97/98(511) | Phosphoethanolamine transferase EptC |
| 14 | *cysE* | complement(12166..12954) | 55.89 | MULTISPECIES: serine O-acetyltransferase [Morganella]( [WP_024473667.1](https://www.ncbi.nlm.nih.gov/protein/WP_024473667.1?report=genbank&log$=protalign&blast_rank=1&RID=ZX5TXX8101R)) | 100/100(266) | Serine acetyltransferase |
| 15 | *gpsA* | complement(12988..14001) | 57.29 | MULTISPECIES: NAD(P)H-dependent glycerol-3-phosphate dehydrogenase [Morganella](  [WP_004240117.1](https://www.ncbi.nlm.nih.gov/protein/WP_004240117.1?report=genbank&log$=protalign&blast_rank=1&RID=ZX5CSD9A01R) ) | 100/100(337) | Glycerol-3-phosphate dehydrogenase [NAD(P)+] |

G6368 (type 11)

| Orf no. | Gene name | Position of gene | G+C content(%) | Similar protein(s), strain(s) (Genbank accession No.) | %Identical/%Similar (total No. of aa) | Putative function of protein |
| --- | --- | --- | --- | --- | --- | --- |
| 1 | *orf1* | 1..897 | 29.65 | phosphotransferase [Morganella morganii]( [WP_087825451.1](https://www.ncbi.nlm.nih.gov/protein/WP_087825451.1?report=genbank&log$=protalign&blast_rank=1&RID=ZXA1XDGN013)) | 100/100(298) | hypothetical protein |
| 2 | *orf2* | 881..1816 | 35.57 | MULTISPECIES: DMT family transporter [Morganella] (  [WP_036417418.1](https://www.ncbi.nlm.nih.gov/protein/WP_036417418.1?report=genbank&log$=protalign&blast_rank=1&RID=ZXA7NCJE013)) | 100/100(311) | hypothetical protein |
| 3 | *orf3* | 1813..2499 | 32.16 | DMT family transporter [Morganella morganii] (  [WP_124537815.1](https://www.ncbi.nlm.nih.gov/protein/WP_124537815.1?report=genbank&log$=protalign&blast_rank=7&RID=ZXA7NCJE013)) | 99/100(311) | UTP--glucose-1-phosphate uridylyltransferase |
| 4 | *tarF* | 2514..3689 | 29.25 | MULTISPECIES: CDP-glycerol glycerophosphotransferase family protein [Morganella] (  [WP_079549407.1](https://www.ncbi.nlm.nih.gov/protein/WP_079549407.1?report=genbank&log$=protalign&blast_rank=1&RID=ZXB17SCP01R)) | 100/100(391) | Teichoic acid poly(glycerol phosphate) polymerase |
| 5 | *GT1* | 3691..4779 | 30.85 | glycosyltransferase [Morganella morganii]( [WP_115983816.1](https://www.ncbi.nlm.nih.gov/protein/WP_115983816.1?report=genbank&log$=protalign&blast_rank=3&RID=ZXBD4KR6013)) | 99/99(362) | GalNAc-alpha-(1->4)-GalNAc-alpha-(1->3)- diNAcBac-PP-undecaprenol alpha-1,4-N-acetyl-D-galactosaminyltransferase |
| 6 | *wzy* | 4769..5845 | 28.22 | EpsG family protein [Morganella morganii]  ( [WP_071888204.1](https://www.ncbi.nlm.nih.gov/protein/WP_071888204.1?report=genbank&log$=protalign&blast_rank=1&RID=ZXBT1R8301R)) | 100/100(358) | hypothetical protein |
| 7 | *tagD* | 5855..6325 | 28.45 | glycerol-3-phosphate cytidylyltransferase [Morganella morganii](  [WP_087696818.1](https://www.ncbi.nlm.nih.gov/protein/WP_087696818.1?report=genbank&log$=protalign&blast_rank=2&RID=ZXBXABYT01R)) | 99/100(156) | Glycerol-3-phosphate cytidylyltransferase |
| 8 | *orf8* | 6325..7326 | 28.94 | sulfotransferase family protein [Morganella morganii]( [WP_064483826.1](https://www.ncbi.nlm.nih.gov/protein/WP_064483826.1?report=genbank&log$=protalign&blast_rank=2&RID=ZXC8B4BB013)) | 99/99(333) | hypothetical protein |
| 9 | *GT2* | 7352..8161 | 29.38 | glycosyltransferase [Morganella morganii](  [WP_087825454.1](https://www.ncbi.nlm.nih.gov/protein/WP_087825454.1?report=genbank&log$=protalign&blast_rank=1&RID=ZXCE1FX0013)) | 100/100(269) | UDP-Gal:alpha-D-GlcNAc-diphosphoundecaprenol beta-1,3-galactosyltransferase |
| 10 | *orf10* | 8166..9311 | 28.79 | hypothetical protein [Morganella morganii] ( [WP_061057585.1](https://www.ncbi.nlm.nih.gov/protein/WP_061057585.1?report=genbank&log$=protalign&blast_rank=1&RID=ZXCJS4FK013)) | 100/100(381) | hypothetical protein |
| 11 | *wzx* | 9308..10756 | 27.46 | oligosaccharide flippase family protein [Morganella morganii](  [WP_061057584.1](https://www.ncbi.nlm.nih.gov/protein/WP_061057584.1?report=genbank&log$=protalign&blast_rank=2&RID=ZXCTH9HW013)) | 99/100(482) | Putative O-antigen transporter |
| 12 | *gnu* | 10774..11724 | 40.48 | NAD-dependent epimerase/dehydratase family protein [Morganella morganii] (  [WP_115983818.1](https://www.ncbi.nlm.nih.gov/protein/WP_115983818.1?report=genbank&log$=protalign&blast_rank=1&RID=ZXCZ0MRF013)) | 100/100(316) | N-acetyl-alpha-D-glucosaminyl-diphospho-ditrans, octacis-undecaprenol 4-epimerase |
| 13 | *trmL* | 11780..12283 | 48.21 | tRNA (uridine(34)/cytosine(34)/5-carboxymethylaminomethyluridine(34)-2'-O)-methyltransferase TrmL [Morganella morganii] (  [WP_032098030.1](https://www.ncbi.nlm.nih.gov/protein/WP_032098030.1?report=genbank&log$=protalign&blast_rank=2&RID=ZXD54MMU013)) | 97/98(167) | tRNA (cytidine(34)-2'-O)-methyltransferase |
| 14 | *orf14* | complement(12355..13128) | 28.03 | hypothetical protein B9Z91_004740 [Morganella morganii subsp. morganii] (  [RNT19063.1](https://www.ncbi.nlm.nih.gov/protein/RNT19063.1?report=genbank&log$=protalign&blast_rank=1&RID=ZXDANBA301R)) | 100/100(257) | hypothetical protein |
| 15 | *cysE* | complement(13331..14134) | 55.47 | serine O-acetyltransferase [Morganella morganii]   ([WP_061057580.1](https://www.ncbi.nlm.nih.gov/protein/WP_061057580.1?report=genbank&log$=protalign&blast_rank=2&RID=ZZXD0XXH013)) | 99/100（267） | Serine acetyltransferase |
| 16 | *gpsA* | complement(14169..15182) | 57.0 | glycerol-3-phosphate dehydrogenase [Morganella morganii]([HAU6097723.1](https://www.ncbi.nlm.nih.gov/protein/HAU6097723.1?report=genbank&log$=protalign&blast_rank=3&RID=ZZXK5K33016) ) | 99/99（337） | Glycerol-3-phosphate dehydrogenase [NAD(P)+] |
