# Supplementary material for: Development of a Molecular Serotyping Scheme for Morganella morganii
Source: Front Microbiol. 2021 Nov 23;12:791165. doi: 10.3389/fmicb.2021.791165 (PMC8649690; doi:10.3389/fmicb.2021.791165)
Supplement: Supplementary file 2 [file Table_2.DOC]

| O-AGC | Strain |
| --- | --- |
| type 1 | AA1; MP63 |
| type3 | 78; VGH116 |
| type5 | AS012489；IS15; NCTC12286; Tanneryeffluent |
| type6 | HE-MDREc28; szy_m28 |
| type7 | MMsCG |
| type8 | 8066; 39876; 171229813; AP69；C135；ICBMmBL-II-04；Jiangxi; MMM_73; NBRC3848; NCTC235; PA18-15564; PA18-16407; SMM01; szy_m16; UMB1297; zy_m3; zy_m16; PA17-10312 |
| type9 | NCTC12358; szy_m22 |
| type11 | CRK0002; CRK0058; FDAARGOS_172; FDAARGOS_438; MM1; MM4; MM190; N18-00103; nx_m63; RD-40764; zy_m2 |

Supplementary Table 2. Allocation of O-AGCs of 42 strains’ genomes under Genbank database that could be assigned to type1 to 11
