# Supplementary material for: Development of a Molecular Serotyping Scheme for Morganella morganii
Source: Front Microbiol. 2021 Nov 23;12:791165. doi: 10.3389/fmicb.2021.791165 (PMC8649690; doi:10.3389/fmicb.2021.791165)
Supplement: Supplementary file 3 [file Table_3.DOC]

| O-AGC | Strain |
| --- | --- |
| temp1 | TW17014; MGYG-HGUT-02513; FAM24091; L3; M.m274; NLAE-zl-C84; B2; B3; szy_m3; szy_m28; KC-Tt-01 |
| temp2 | MRSN22709; Colony456 |
| temp3 | NCTC232; L241; E042; ATCC25830; KT; MGYG-HGUT-02512; NCTC12028; SCsl21 |
| temp4 | 640_MMOR; AR_0057; AR_0133; F675; szy_m40; EH8; FDAARGOS_365 |
| temp5 | MM51087; MM50821; MM46903; MMM_59 |
| temp6 | NCTC12289; 340; 4601; DG56-16; SCsl4；CQ-M7；FDAARGOS_63；M006；GN28；PA18-25921 |

Supplementary Table 3. Allocation of putative novel O-AGCs extracted from 41 strains’ genomes deposited under Genbank database
