# Supplementary figures and images for: Development of a Molecular Serotyping Scheme for Morganella morganii
Source: Front Microbiol. 2021 Nov 23;12:791165. doi: 10.3389/fmicb.2021.791165 (PMC8649690; doi:10.3389/fmicb.2021.791165)

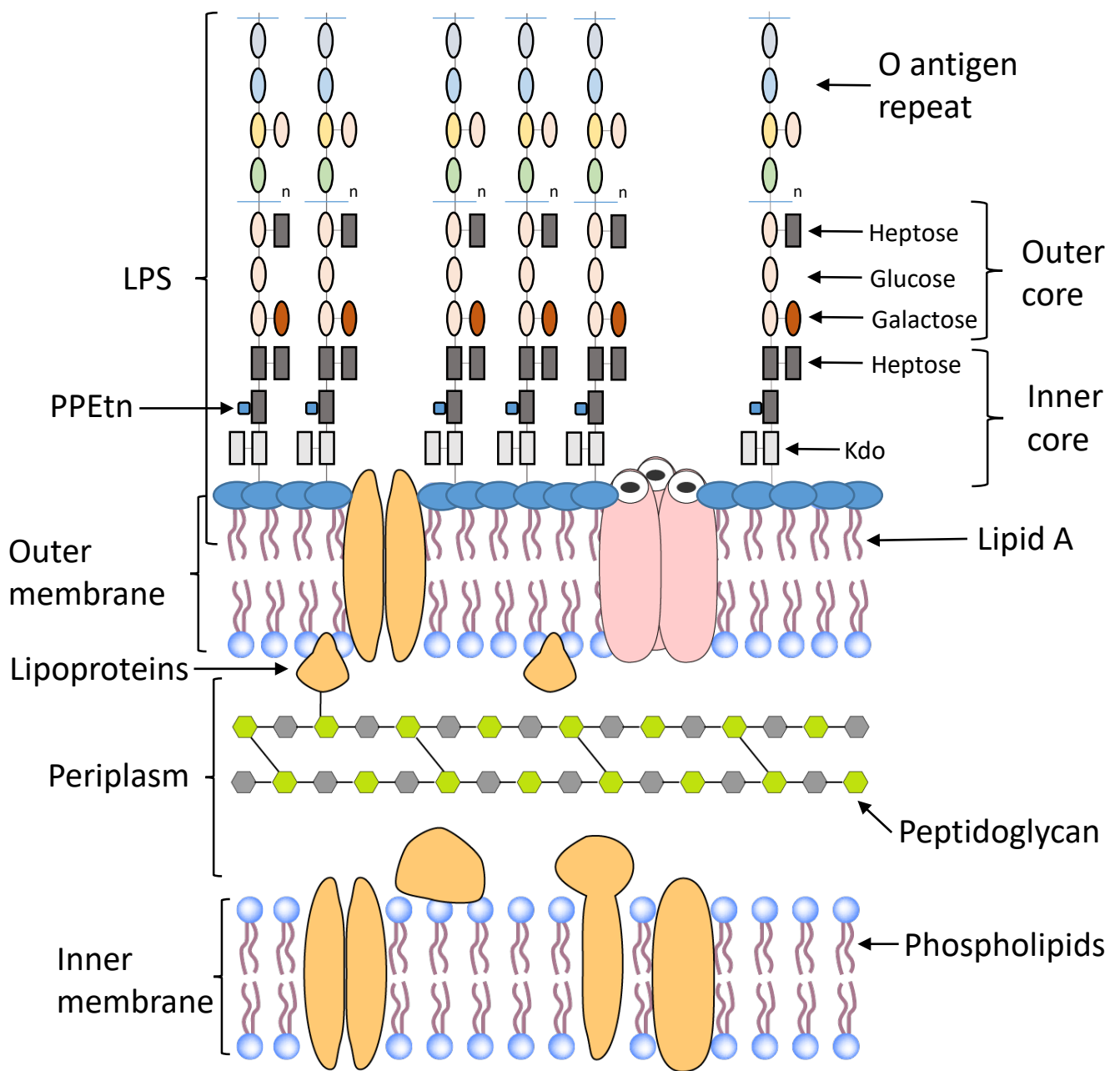

Supplementary Figure 1. A model structure of lipopolysaccharide (LPS)

Supplement: Supplementary file 4 [file Data_Sheet_1.PDF]
